# Primary mono- and bis-sulfonamides obtained via regiospecific sulfochlorination of N-arylpyrazoles: inhibition profile against a panel of human carbonic anhydrases

**DOI:** 10.1080/14756366.2017.1344236

**Published:** 2017-07-18

**Authors:** Mikhail Krasavin, Mikhail Korsakov, Oksana Ronzhina, Tiziano Tuccinardi, Stanislav Kalinin, Muhammet Tanç, Claudiu T. Supuran

**Affiliations:** a Saint Petersburg State University, Saint Petersburg, Russian Federation;; b The Ushinsky Yaroslavl State Pedagogical University, Yaroslavl, Russian Federation;; c Department of Pharmacy, University of Pisa, Pisa, Italy;; d Department of Neurofarba, Universita degli Studi di Firenze, Florence, Italy

**Keywords:** Carbonic anhydrases, isoform selectivity, direct sulfochlorination, mono-sulfonamides, bis-sulfonamides, chemoselectivity

## Abstract

A diverse set of mono- and bis-sulfonamide was obtained via a direct, chemoselective sulfochlorination of readily available yet hitherto unexplored N-arylpyrazole template. Biochemical profiling of compounds thus obtained against a panel of human carbonic anhydrases (*h*CA I, *h*CA II, *h*CA IV and *h*CA VII) revealed a number of leads that are promising from the isoform selectivity prospective and exhibit potent inhibition profile (from nanomolar to micromolar range). The observed SAR trends have been rationalized by *in silico* docking of selected compounds into the active site of all four isoforms. The results reported in this paper clearly attest to the power of direct sulfochlorination as the means to create carbonic anhydrase focused sets in order to identify isoform selective inhibitors of closely related enzymes.

## Graphical Abstract

**Figure:**
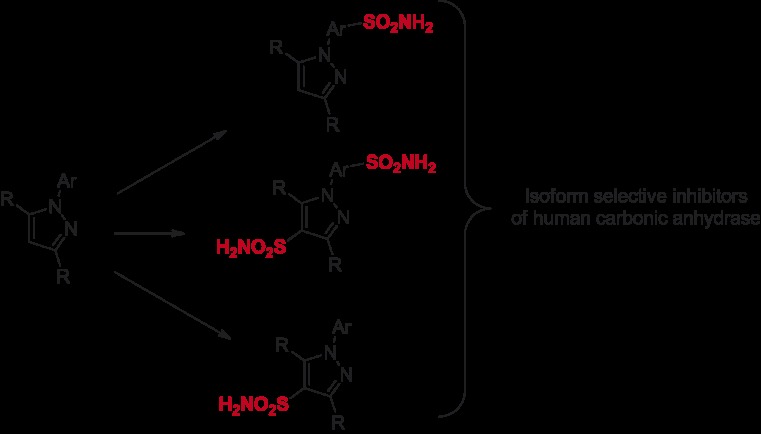

## Introduction

**Figure 1.**
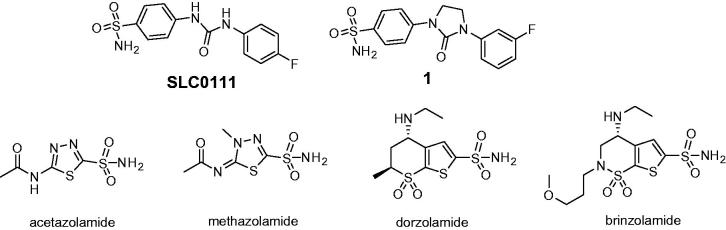
Advanced and clinically used *h*CA inhibitors.

Spiking a carbo- or heterocyclic compound with a primary sulfonamide group has manifested itself a remarkably efficient strategy to render the molecule somewhat inhibitory toward human carbonic anhydrase (*h*CA) due to prosthetic Zinc binding by that group (with numerous limitations currently known to that approach) – and, ultimately, determine which of the resulting molecules will have (or at least show tendencies to have) favourable isoform-inhibitory profiles^1^. These much-needed, early leads can subsequently scrutinized from a structural viewpoint, advanced into clinical status (such as SCL-0111^2^) or gain a more evolutionary look (such as compound **1**^3^ developed for treatment of cancer metastases). It’s been a long-standing dogma that the currently available landscape of drugs acting via pan-isoform inhibition mechanism (e.g. acetazolamide, methazolmide, dorzolamide and brinzolamide) are all efficacious but suboptimal in terms of inhibiting several isoforms at the same time (Figure 1)^4^.

**Figure 2.**
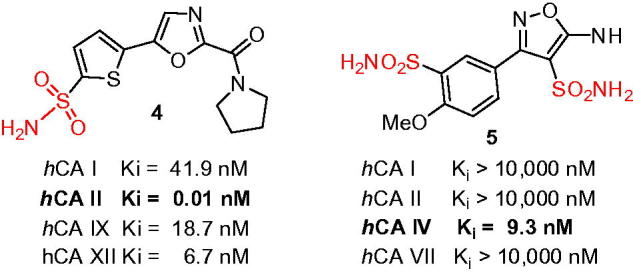
Isoform-selective CAIs derivable by direct sulfochlorination approach.

**Figure 3.**
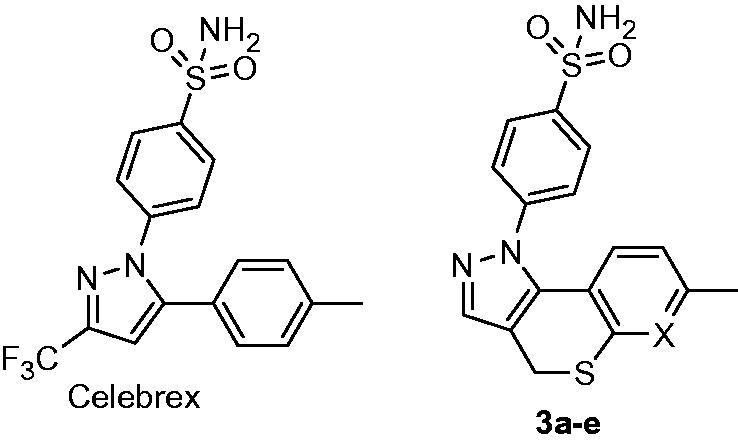
Celebrex and its tricyclic constrained versions **3a–e** reported earlier.

They are poor research tools on the one hand (i.e. any attempt to link the biology perturbed by them to reality would be a dicey undertaking). On the other hand, cleaner isoform selectivity of a therapeutic agent has always been a holy grail of pharma companies: such drugs are considered to have fewer off-targets, which usually means less side-effects^5^. There is one more aspect as to the isoform *h*CA selectivity worth mentioning here, perhaps even more puzzling. The localization of various *h*CA isoforms within the cell is uneven and some are more or become more important than others, especially when the disease strikes (Figure 2). Take *h*CA IX that can is expressed on a cell membrane and became the main defenders of cells in tumors^6^. Clearly, we need tools to tackle *h*CA isoform selectivity. One such tool to use would be chemical diversity and, indeed, numerous chemically diverse series of carbonic anhydrase inhibitors (CAIs) have been profiled today^4^. The power of multicomponent chemistry to deliver CAIs has been relatively underutilized today as was recently reviewed^7^ and we are currently working to fill this void. Herein, we report a somewhat intermittent approach, namely, a systematic conversion of a set of N-arylpyrazines **2a–t** into tractable and SAR-informative set of primary mono- and bis-sulfomamides. The substrates are relevant to, among other pharmacologically sound molecules, known blockbuster antipyretic Celebrex as well as tricyclic congeners **3a–e** earlier reported by Marini (Figure 3)^8^.

**Figure 4.**
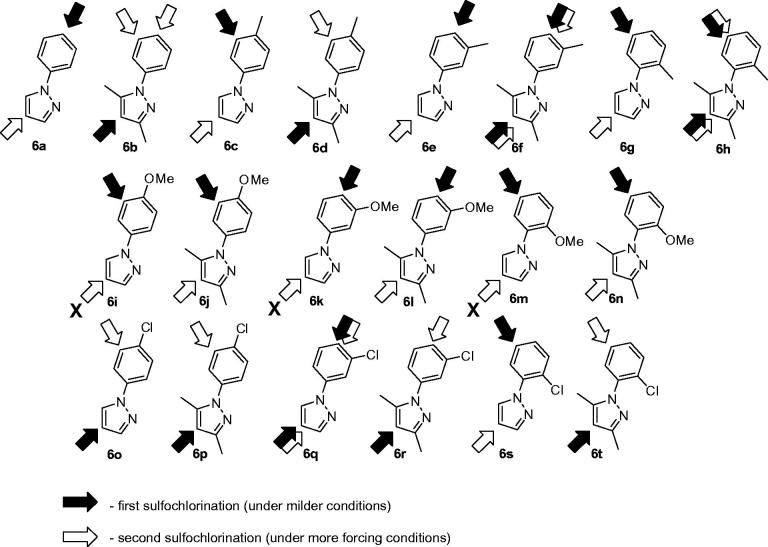
*N*-Arylpyrazole substrates **6a-t** investigated in direct mono- and bis-sulfochlorination reactions (regiochemistry established for the respective mono- and bis-sulfonamides).

Moreover, through some less systematic approach, the direct sulfochlorination of (hetero)aromatics has very recently given rise to: (i) 5-thienyl-1,3-oxazolecarboxamides **4** (where a remarkable potency toward *h*CA II with *K*_i_ = 0.01 nM was achieved)^9^ and (ii) a series of isoxazole bis-sulfonamides, exemplified by **5** clearly offering an alternative ZBG binging mode and a remarkable *K*_i_ of 9.4 nM against *h*CA IV, an extremely rare *h*CA to target with such a potency and selectivity (Figure 4)^10^.

These isolated and nonetheless successful results, prompt us to undertake a direct sulfochlorination approach to produce compounds which would not only provide a wealthy entry into the realm of CAI but also provide the reader with an easy-to-read compendium of methods on direct shofochlorination of Celebrex-like N-arylpyrazoles.

## Materials and methods

### Chemical syntheses – general

All reactions were carried out in oven-dried glassware in atmosphere of nitrogen. Melting points were measured with a Buchi В-520 melting point apparatus and are uncorrected. Thin-layer chromatography was carried out on Silufol UV-254 silica gel plates using an appropriate mixture of ethyl acetate and hexane. Compounds were visualized with short-wavelength UV light. ^1^H NMR and ^13^C NMR spectra were recorded on Bruker MSL-300 spectrometers in DMSO-d_6_ using TMS as an internal standard. Elemental analyses were obtained at Research Institute for Chemical Crop Protection (Moscow, Russia) using Carlo Erba Strumentazione 1106 analyser. Mass spectra were recorded using Shimadzu LCMS-2020 system with electron impact (EI) ionization. All and reagents and solvents were obtained from commercial sources and used without purification.

### General procedure 1 (GP1): regiochemically unambiguous preparation of monosulfonamides 7–8, not requiring chromatographic separation regioisomers of sulfonyl chlorides 10–11

To a well-stirred ice-cold mixture of 6.76 g (58.1 mmol) or of chlorosulfonic acid and 0.76 g (6.4 mmol) thionyl chloride was added, in small portions, an appropriate precursor **6** (5.8 mmol). The mixture was heated at the temperature and for the period of time indicated in Tables 1–3. The reaction mixture was cooled to ambient temperature and poured over ice (250 g). The resulting mixture was extracted with chloroform (100 ml). The organic layer was separated, washed with water (200 ml), 5% aqueous K_2_CO_3_, dried over anhydrous CaCl_2_ and filtered through a short plug of silica. The volatiles were removed *in vacuo* and the residue dissolved in acetone (15 ml) and the resulting clear solution was treated with 25% aqueous ammonia solution (29.0 mmol). The resulting mixture was heated at 50 °C for 30 min, concentrated *in vacuo* and the residue was dispersed in water (50 ml) the resulting fine precipitate was separated by filtration, washed with more water (100 ml) and air dried. Crystallization from isopropyl alcohol provided analytically pure mono- (**7–8**) and bis-sulfamides (**9**) in yields indicated.

**Table 1.** Inhibitory profile of mono-sulfonamides **7a–o** against four *h*CA isoforms.
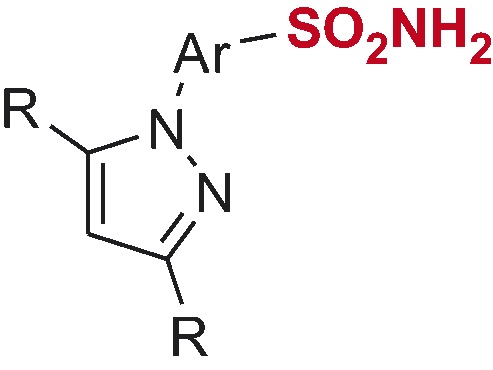

			Sulfochlorination step		*h*CA *K*_i_ (μM)			
Compound	Substrate **6**	Structure	*T* (°C)	Time (h)	*h*CA I	*h*CA II	*h*CA IV	*h*CA VII
**7a**	**6a**		70	6	4.78	0.072	>10.0	0.80
**7b**	**6c**		70	3	>10.0	>10.0	0.56	>10.0
**7c**	**6f**		20	22	0.26	0.004	0.033	0.040
**7d**	**6e**		70	3	0.096	0.008	3.42	0.014
**7e**	**6h**		20	22	4.21	0.381	>10.0	0.194
**7f**	**6g**		70	3	1.23	0.059	>10.0	0.059
**7g**	**6j**		20	20	>10.0	>10.0	>10.0	>10.0
**7h**	**6i**		20	24	>10.0	>10.0	>10.0	>10.0
**7i**	**6l**		20	1	8.85	>10.0	>10.0	>10.0
**7j**	**6k**		20	24	>10.0	>10.0	>10.0	0.387
**7k**	**6n**		10	1	>10.0	>10.0	>10.0	>10.0
**7l**	**6m**		20	24	>10.0	6.65	>10.0	>10.0
**7m**	**6q**		80	3	0.066	0.085	0.086	0.328
**7n**	**6s**		90	5	3.83	0.001	0.004	0.009
**7o**	–		–	–	0.462	0.004	0.084	0.017
Acetazolamide					0.25	0.012	0.074	0.003

**Table 2.** Inhibitory profile of mono-sulfonamides **8a–i** against four *h*CA isoforms.
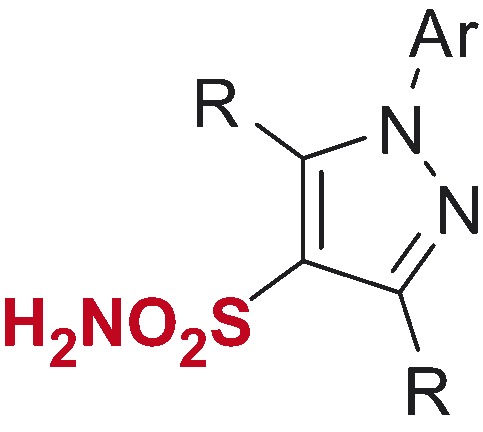

			Sulfochlorination		*h*CA *K*_i_ (μM)			
Compound	Substrate **6**	Structure	*T* (°C)	Time (h)	*h*CA I	*h*CA II	*h*CA IV	*h*CA VII
**8a**	**6p**		70	1	0.76	5.33	>10.0	>10.0
**8b**	**6b**		70	1	0.54	0.28	>10.0	0.53
**8c**	**6d**		20	20	0.76	0.74	>10.0	>10.0
**8d**	**6f**		20	22	0.27	0.24	>10.0	0.94
**8e**	**6h**		20	22	0.60	0.091	>10.0	>10.0
**8f**	**6o**		90	4	0.19	0.082	7.06	>10.0
**8g**	**6r**		70	5	0.94	1.08	9.49	0.46
**8h**	**6q**		80	3	0.54	0.14	3.74	0.24
**8i**	**6t**		70	10	0.62	2.64	7.19	>10.0
Acetazolamide					0.25	0.012	0.074	0.003

**Table 3.** Inhibitory profile of mono-sulfonamides **9a–s** against four *h*CA isoforms.
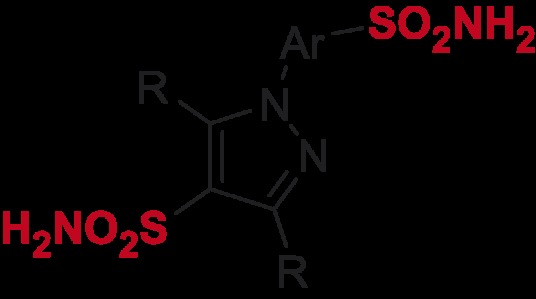

			Sulfochlorination		*h*CA *K*_i_ (μM)			
Compound	Substrate **xx**	Structure	*T* (°C)	Time (h)	*h*CA I	*h*CA II	*h*CA IV	*h*CA VII
**9a**	**6b**		70	7	0.50	0.021	0.065	0.26
**9b**	**6b**		70	7	0.33	0.005	0.111	0.035
**9c**	**6a**		100	48	0.20	0.002	0.025	0.041
**9d**	**6d**		70	7	>10.0	>10.0	0.40	>10.0
**9e**	**6c**		100	20	0.30	0.004	0.24	0.068
**9f**	**6f**		70	7	0.47	0.002	0.022	0.019
**9g**	**6e**		100	20	0.10	0.049	5.80	0.005
**9h**	**6h**		70	7	0.87	>10.0	>10.0	>10.0
**9i**	**6g**		100	20	0.55	0.030	7.86	0.014
**9j**	**6j**		70	4	0.95	0.96	>10.0	0.33
**9k**	**6l**		70	4	4.65	0.61	>10.0	0.17
**9l**	**6n**		70	4	6.80	0.30	0.95	>10.0
**9m**	**6p**		100	24	0.89	0.040	0.077	0.65
**9n**	**6o**		120	10	0.62	0.024	0.074	0.099
**9o**	**6r**		100	24	0.16	0.008	0.93	0.71
**9p**	**6q**		120	20	3.08	0.008	0.075	0.48
**9q**	**6t**		100	24	2.12	0.025	4.38	>10.0
**9r**	**6s**		120	20	0.55	0.005	0.022	0.60
Acetazolamide					0.25	0.012	0.074	0.003

#### 4-(^1^H-pyrazol-1-yl)benzenesulfonamide (7a)

Prepared from **6a** according to GP1; white solid, m.p. 323–325°C (*i*-PrOH), yield 73%; ^1^H NMR (300 MHz, DMSO-d_6_) *δ* ppm 11.44 (br. s., 2H, SO_2_NH_2_), 8.50 (d, *J* = 2.3 Hz, 1H, H_pyrazole_), 7.82 (d, *J* = 8.6 Hz, 2H, H_Ar_), 7.74 (d, *J* = 1.4 Hz, 1H, H_pyrazole_), 7.72 (d, *J* = 8.6 Hz, 2H, H_Ar_), 6.56 (dd, *J*_1_=2.3 Hz, *J*_2_=1.4 Hz, 1H, H_pyrazole_); ^13^C NMR (75 MHz, DMSO-d_6_) *δ* ppm 146.0, 141.6, 140.1, 128.3, 127.3, 118.0, 108.5; LC/MS (ESI^+^): *m/z* [M + H]^+^ 224.3; Anal. calcd for C_9_H_9_N_3_O_2_S (223.25): C, 48.42; H, 4.06; N, 18.82; S, 14.36; found: C, 48.39; H, 4.06; N, 18.84; S, 14.37.

#### 2-Methyl-5-(^1^H-pyrazol-1-yl)benzenesulfonamide (7b)

Prepared from **6c** according to GP1; yellow solid, m.p. 147–149°C (*i*-PrOH), yield 68%. ^1^H NMR (400 MHz, DMSO-d_6_) *δ* ppm 8.38 (d, *J* = 2.4 Hz, 1H, H_pyrazole_), 8.17 (d, *J*_4–6_ = 2.4 Hz, 1H, 6-H_A_), 7.78 (br. s., 2H, SO_2_NH_2_), 7.71 (d, *J* = 1.5 Hz, 1H, H_pyrazole_), 7.63 (dd, *J*_3–4_ = 8.2, Hz, *J*_4–6_ = 2.4 Hz, 1H, 4-H_Ar_), 7.25 (d, *J*_3–4_ = 8.2 Hz, 1H, 3-H_Ar_), 6.51 (dd, *J*_1_=2.4 Hz, *J*_2_=1.5 Hz, 1H, H_pyrazole_), 2.54 (s, 3H, Ar-CH_3_). ^13^C NMR (75 MHz, DMSO-d_6_) *δ* ppm 147.5, 141.1, 137.3, 133.9, 132.2, 127.9, 118.9, 117.5, 108.1, 19.9. LC/MS (ESI^+^): *m/z* [M + H]^+^ 238.3. Anal. calcd for C_10_H_11_N_3_O_2_S (237.28): C, 50.62; H, 4.67; N, 17.71; S, 13.51; found: C, 50.59; H, 4.68; N, 17.75; S, 13.52.

#### 2-Methyl-4-(^1^H-pyrazol-1-yl)benzenesulfonamide (7d)

Prepared from **6e** according to GP1; white solid, m.p. 161–163 °C (*i*-PrOH), yield 66%. ^1^H NMR (400 MHz, DMSO-d_6_) *δ* ppm 8.59 (d, *J* = 2.4 Hz, 1H, H_pyrazole_), 7.94 (d, *J*_3–4_ = 8.6 Hz, 1H, 3-H_Ar_), 7.89 (d, *J*_3–5_ = 2.0 Hz, 1H, 3-H_A_), 7.82 (dd, *J*_5–6_ = 8.6 Hz, *J*_3–5_ = 2.0 Hz, 1H, 5-H_Ar_), 7.80 (d, *J* = 1.6 Hz, 1H, H_pyrazole_), 7.42 (br. s., 2H, SO_2_NH_2_), 6.59 (dd, *J*_1_=2.4 Hz, *J*_2_=1.6 Hz, 1H, H_pyrazole_), 2.66 (s, 3H, CH_3_). ^13^C NMR (75 MHz, DMSO-d_6_) *δ* ppm 142.2, 142.0, 139.9, 138.3, 129.2, 128.7, 121.6, 115.6, 108.9, 20.4. LC/MS (ESI^+^): *m/z* [M + H]^+^ 238.3. Anal. calcd for C_10_H_11_N_3_O_2_S (237.28): C, 50.62; H, 4.67; N, 17.71; S, 13.51; found: C, 50.57; H, 4.67; N, 17.68; S, 13.52.

#### 4-Methyl-3-^1^H-pyrazol-1-yl)benzenesulfonamide (7f)

Prepared from **6 g** according to GP1; light brown solid, m.p. 143–145 °C (*i*-PrOH), yield 61%. ^1^H NMR (400 MHz, DMSO-d_6_) *δ* ppm 8.12 (d, *J* = 2.2 Hz, 1H, 5-H_pyrazole_), 7.78 (m, 3H, 3-H_pyrazole_, 2-H_Ar_, 6-H_Ar_), 7.60 (d, *J*_5–6_ = 7.8 Hz, 1H, 5-H_Ar_), 7.42 (s, 2H, SO_2_NH_2_), 6.56 (m, 1H, 4-H_pyrazole_), 2.29 (s, 3H, CH_3_); ^13^C NMR (75 MHz, DMSO-d_6_) *δ* ppm 143.2, 141.1, 140.1, 137.1, 132.4, 131.9, 125.3, 123.5, 107.4, 18.5. LC/MS (ESI^+^): *m/z* [M + H]^+^ 238.3. Anal. calcd for C_10_H_11_N_3_O_2_S (237.28): C, 50.62; H, 4.67; N, 17.71; S, 13.51; found: C, 50.57; H, 4.67; N, 17.68; S, 13.52.

#### 5-(3,5-Dimethyl-^1^H-pyrazol-1-yl)-2-methoxybenzenesulfonamide (7g)

Prepared from **6j** according to GP1; white solid, m.p. 195–197°C (*i*-PrOH), yield 78%. ^1^H NMR (400 MHz, DMSO-d_6_) *δ* ppm 7.76 (d, *J*_4–6_ = 2.7 Hz, 1H, 6-H_Ar_), 7.44 (dd, *J*_3–4_ = 8.6 Hz, *J*_4–6_ = 2.7 Hz, 1H, 4-H_Ar_), 7.09 (d, *J*_3–4_ = 8.6 Hz, 1H, 3-H_Ar_), 6.18 (s, 1H, H_pyrazole_), 5.03 (br. s., 2H, SO_2_NH_2_, H_2_O), 3.82 (s, 3H, OCH_3_), 2.22 (s, 6H, 2xCH_3_); ^13^C NMR (75 MHz, DMSO-d_6_) *δ* ppm 156.8, 147.1, 142.3, 136.8, 128.9, 127.6, 126.1, 112.9, 107.5, 56.4, 12.8, 12.1. LC/MS (ESI^+^): *m/z* [M + H]^+^ 282.3. Anal. calcd for C_12_H_15_N_3_O_3_S (281.33): C, 51.23; H, 5.37; N, 14.94; S, 11.40; found: C, 51.17; H, 5.38; N, 14.91; S, 11.41.

#### 2-Methoxy-5-^1^H-pyrazol-1-yl)benzenesulfonamide (7h)

Prepared from **6i** according to GP1; white solid, m.p. 138–140 °C (*i*-PrOH), yield 63%. ^1^H NMR (300 MHz, DMSO-d_6_) *δ* ppm 8.47 (d, *J* = 2.0 Hz, 1H, H_pyrazole_), 8.18 (d, *J*_4–6_ = 2.7 Hz, 1H, 6-H_Ar_), 8.00 (dd, *J*_3–4_ = 8.9 Hz, *J*_4–6_ = 2.7 Hz, 1H, 4-H_Ar_), 7.74 (s, 1H, H_pyrazole_), 7.33 (d, *J*_3–4_ = 8.9 Hz, 1H, 3-H_Ar_), 7.23 (s, 2H, SO_2_NH_2_), 6.54 (m, 1H, H_pyrazole_), 3.95 (s, 3H, OCH_3_). ^13^C NMR (75 MHz, DMSO-d_6_) *δ* ppm 155.1, 140.8, 136.2, 132.5, 127.9, 121.1, 120.0, 113.5, 107.8, 56.4. LC/MS (ESI^+^): *m/z* [M + H]^+^ 254.3. Anal. calcd for C_10_H_11_N_3_O_3_S (253.28): C, 47.42; H, 4.38; N, 16.59; S, 12.66; found: C, 47.40; H, 4.38; N, 15.62; S, 12.67.

#### 4-(3,5-Dimethyl-^1^H-pyrazol-1-yl)-2-methoxybenzenesulfonamide (7i)

Prepared from **6l** according to GP1; yellow solid, m.p. 156–158 °C (*i*-PrOH), yield 72%; ^1^H NMR (400 MHz, DMSO-d_6_) *δ* ppm 11.95 (br. s., 2H, SO_2_NH_2_, H_2_O),7.76 (d, *J*_5–6_ = 8.2 Hz, 1H, 6-H_Ar_), 7.08 (d, *J*_3–5_ = 1.8 Hz, 1H, 3-H_Ar_), 6.99 (dd, *J*_5–6_ = 8.2 Hz, *J*_3–5_ = 1.8 Hz, 1H, 5-H_Ar_), 6.15 (s, 1H, H_pyrazole_), 3.79 (s, 3H, OCH_3_), 2.32 (s, 3H, CH_3_), 2.24 (m, 3H, CH_3_). ^13^C NMR (75 MHz, DMSO-d_6_) *δ* ppm 157.0, 149.2, 144.3, 140.2, 129.8, 128.8, 114.8, 108.7, 108.7, 56.9, 13.7, 12.9. LC/MS (ESI^+^): *m/z* [M + H]^+^ 282.3. Anal. calcd for C_12_H_15_N_3_O_3_S (281.33): C, 51.23; H, 5.37; N, 14.94; S, 11.40; found: C, 51.21; H, 5.37; N, 14.95; S, 11.41.

#### 2-Methoxy-4-(^1^H-pyrazol-1-yl)benzenesulfonamide (7j)

Prepared from **6k** according to GP1; white solid, m.p. 221–223°C (*i*-PrOH), yield 64%. ^1^H NMR (400 MHz, DMSO-d_6_) *δ* ppm 8.66 (d, *J* = 2.4 Hz, 1H, H_pyrazole_), 7.81 (d, *J*_5–6_ = 8.4 Hz, 1H, 6-H_Ar_), 7.81 (d, *J* = 1.0 Hz, 1H, H_pyrazole_), 7.63 (d, *J*_3–5_ = 1.6 Hz, 1H, 3-H_Ar_), 7.54 (dd, *J*_5–6_ = 8.4 Hz, *J*_3–5_ = 1.6 Hz, 1H, 5-H_Ar_), 7.12 (s, 2H, SO_2_NH_2_), 6.61 (m, 1H, H_pyrazole_), 4.00 (s, 3H, OCH_3_). ^13^C NMR (75 MHz, DMSO-d_6_) *δ* ppm 157.5, 144.0, 142.3, 129.6, 129.3, 129.0, 109.5, 109.0, 103.1, 56.9. LC/MS (ESI^+^): *m/z* [M + H]^+^ 254.3. Anal. calcd for C_10_H_11_N_3_O_3_S (253.28): C, 47.42; H, 4.38; N, 16.59; S, 12.66; found: C, 47.38; H, 4.38; N, 15.58; S, 12.66.

#### 3-(3,5-Dimethyl-^1^H-pyrazol-1-yl)-4-methoxybenzenesulfonamide (7k)

Prepared from **6n** according to GP1; white solid, m. p. 167–170 °C (*i*-PrOH); ^1^H NMR (400 MHz, DMSO-d_6_) *δ* ppm 7.64 (dd, *J*_5–6_ = 8.6 Hz, *J*_2–6_ = 2.2 Hz, 1H, 6-H_Ar_), 7.41 (d, *J*_2–6_ = 2.2 Hz, 1H, 2-H_Ar_), 7.14 (d, *J*_5–6_ = 8.6 Hz, 1H, 5-H_Ar_), 7.11 (br. s., 2H, SO_2_NH_2_), 5.97 (s, 1H, H_pyrazole_), 3.78 (s, 3H, OCH_3_), 2.15 (s, 3H, CH_3_), 1.99 (s, 3H, CH_3_). ^13^C NMR (75 MHz, DMSO-d_6_) *δ* ppm 154.4, 148.0, 141.3, 127.6, 127.5, 127.5, 126.7, 112.1, 105.7, 56.4, 13.8, 11.3. LC/MS (ESI^+^): *m/z* [M + H]^+^ 282.3. Anal. calcd for C_12_H_15_N_3_O_3_S (281.33): C, 51.23; H, 5.37; N, 14.94; S, 11.40; found: C, 51.20; H, 5.37; N, 14.96; S, 11.41.

#### 4-Methoxy-3-(^1^H-pyrazol-1-yl)benzenesulfonamide (7l)

Prepared from **6m** according to GP1; white solid, m.p. 255–257 °C (*i*-PrOH), yield 61%. ^1^H NMR (400 MHz, DMSO-d_6_) *δ* ppm 9.40 (br. s., 2H, SO_2_NH_2_, H_2_O), 8.17 (d, *J* = 2.45 Hz, 1H, H_pyrazole_), 7.86 (d, *J*_2–6_ = 2.2 Hz, 1H, 2-H_Ar_), 7.70 (d, *J* = 1.7 Hz, 1H, H_pyrazole_), 7.56 (dd, *J*_5–6_ = 8.6 Hz, *J*_2–6_ = 2.2 Hz, 1H, 6-H_Ar_), 7.19 (d, *J*_5–6_ = 8.6 Hz, 1H, 5-H_Ar_), 6.47 (m, 1H, H_pyrazole_), 3.87 (s, 3H, OCH_3_). ^13^C NMR (75 MHz, DMSO-d_6_) *δ* ppm 151.42, 141.22, 140.30, 132.30, 128.39, 125.80, 122.75, 112.60, 106.98, 56.73. LC/MS (ESI^+^): *m/z* [M + H]^+^ 254.3. Anal. calcd for C_10_H_11_N_3_O_3_S (253.28): C, 47.42; H, 4.38; N, 16.59; S, 12.66; found: C, 47.31; H, 4.38; N, 15.64; S, 12.68.

#### 4-Chloro-3-(^1^H-pyrazol-1-yl)benzenesulfonamide (7n)

Prepared from **6s** according to GP1; off-white solid, m.p. 204–206 °C (*i*-PrOH); ^1^H NMR (400 MHz, DMSO-d_6_) *δ* ppm 8.26 (d, *J* = 2.2 Hz, 1H, H_pyrazole_), 8.00 (d, *J*_2–6_ = 1.7 Hz, 1H, 2-H_Ar_), 7.91 (d, *J*_5–6_ = 8.3 Hz, 1H, 5-H_Ar_), 7.86 (dd, *J*_5–6_ = 8.3 Hz, *J*_2–6_ = 1.7 Hz, 1H, 6-H_Ar_), 7.83 (d, *J* = 1.5 Hz, 1H, H_pyrazole_), 7.60 (s, 2H, SO_2_NH_2_), 6.59 (m, 1H, H_pyrazole_). ^13^C NMR (75 MHz, DMSO-d_6_) *δ* ppm 144.4, 141.8, 138.4, 132.7, 131.9, 131.1, 126.7, 125.4, 107.7. LC/MS (ESI^+^): *m/z* [M + H]^+^ 258.7. Anal. calcd for C_9_H_8_ClN_3_O_2_S (257.70): C, 41.95; H, 3.13; N, 16.31; S, 12.44; found: C, 41.91; H, 3.13; N, 16.27; S, 12.46.

#### 1-(4-Chlorophenyl)-3,5-dimethyl-^1^H-pyrazole-4-sulfonamide (8a)

Prepared from **6p** according to GP1; white solid, m.p. 164–166 °C (*i*-PrOH), yield 64%; ^1^H NMR (400 MHz, DMSO-d_6_) *δ* ppm 7.60 (d, *J* = 9.0 Hz, 2H, H_Ar_), 7.54 (d, *J* = 9.0 Hz, 2H, H_Ar_), 7.24 (s, 2H, SO_2_NH_2_), 2.42 (s, 3H, CH_3_), 2.36 (s, 3H, CH_3_). ^13^C NMR (75 MHz, DMSO-d_6_) *δ* ppm 147.5, 141.0, 137.6, 133.4, 129.8, 127.5, 122.2, 66.8, 13.4, 11.9. LC/MS (ESI^+^): *m/z* [M + H]^+^ 286.7. Anal. calcd for C_11_H_12_ClN_3_O_2_S (285.75): C, 46.24; H, 4.23; N, 14.71; S, 11.22; found: C, 46.20; H, 4.23; N, 14.75; S, 11.23.

#### 3,5-Dimethyl-1-phenyl-^1^H-pyrazole-4-sulfonamide (8b)

Prepared from **6b** according to GP1; white solid, m.p. 161–163 °C (*i*-PrOH), yield 67%; ^1^H NMR (400 MHz, DMSO-d_6_) *δ* ppm 7.55 (m, 2H, H_Ar_), 7.49 (m, 3H, H_Ar_), 7.22 (s, 2H, SO_2_NH_2_), 2.41 (s, 3H, CH_3_), 2.36 (s, 3H, CH_3_); ^13^C NMR (75 MHz, DMSO-d_6_) *δ* ppm 147.2, 140.8, 138.7, 129.8, 128.9, 125.9, 121.8, 13.5, 12.0. LC/MS (ESI^+^): *m/z* [M + H]^+^ 252.3. Anal. calcd for C_11_H_13_N_3_O_2_S (251.31): C, 52.57; H, 5.21; N, 16.72; S, 12.76; found: C, 52.54; H, 5.22; N, 16.75; S, 12.78.

#### 3,5-Dimethyl-1-(4-methylphenyl)-^1^H-pyrazole-4-sulfonamide (8c)

Prepared from **6d** according to GP1; white solid, m.p. 171–174 °C (*i*-PrOH), yield 63%; ^1^H NMR (400 MHz, DMSO-d_6_) *δ* ppm 7.35 (m, 4H, H_Ar_), 7.20 (s, 2H, SO_2_NH_2_), 2.39 (s, 3H, CH_3_), 2.38 (s, 3H, CH_3_), 2.35 (s, 3H, CH_3_); ^13^C NMR (75 MHz, DMSO-d_6_) *δ* ppm 147.0, 140.7, 138.6, 136.4, 130.2, 125.7, 121.7, 21.1, 13.4, 11.9. LC/MS (ESI^+^): *m/z* [M + H]^+^ 266.3. Anal. calcd for C_12_H_15_N_3_O_2_S (265.34): C, 54.32; H, 5.70; N, 15.84; S, 12.08; found: C, 54.30; H, 5.70; N, 15.82; S, 12.10.

#### 1-(4-Chlorophenyl)-^1^H-pyrazole-4-sulfonamide (8f)

Prepared from **6o** according to GP1; off-white solid, m.p. 147–150 °C (*i*-PrOH); ^1^H NMR (300 MHz, DMSO-d_6_) *δ* ppm 8.99 (s, 1H, H_pyrazole_), 8.04 (s, 1H, H_pyrazole_), 7.94 (d, *J* = 8.9 Hz, 2H, H_Ar_), 7.59 (d, *J* = 8.9 Hz, 1H, H_Ar_), 7.43 (s, 2H, SO_2_NH_2_); ^13^C NMR (75 MHz, DMSO-d_6_) *δ* ppm 139.5, 138.1, 132.1, 130.1, 129.6, 128.9, 121.3. LC/MS (ESI^+^): *m/z* [M + H]^+^ 258.7. Anal. calcd for C_9_H_8_ClN_3_O_2_S (257.70): C, 41.95; H, 3.13; N, 16.31; S, 12.44; found: C, 41.89; H, 3.13; N, 16.28; S, 12.45.

#### 1-(3-Chlorophenyl)-3,5-dimethyl-^1^H-pyrazole-4-sulfonamide (8g)

Prepared from **6r** according to GP1; white solid, m.p. 182–184 °C (*i*-PrOH), yield 54%. ^1^H NMR (400 MHz, DMSO-d_6_) *δ* ppm 7.56 (m, 4H, H_Ar_), 7.25 (s, 2H, SO_2_NH_2_), 2.45 (s, 3H, CH_3_), 2.37 (s, 3H, CH_3_). ^13^C NMR (75 MHz, DMSO-d_6_) *δ* ppm 147.6, 141.1, 139.9, 134.0, 131.3, 128.9, 125.6, 124.5, 122.3, 13.5, 11.9. LC/MS (ESI^+^): *m/z* [M + H]^+^ 286.7. Anal. calcd for C_11_H_12_ClN_3_O_2_S (285.75): C, 46.24; H, 4.23; N, 14.71; S, 11.22; found: C, 46.18; H, 4.24; N, 14.69; S, 11.24.

#### 1-(2-Chlorophenyl)-3,5-dimethyl-^1^H-pyrazole-4-sulfonamide (8i)

Prepared from **6t** according to GP1; white solid, m.p. 204–206°C (AcOEt), yield 56%. ^1^H NMR (300 MHz, DMSO-d_6_) *δ* ppm 7.73 (d, *J* = 7.60 Hz, 1H, H_Ar_), 7.59 (m, 3H, H_Ar_), 7.27 (s, 2H, SO_2_NH_2_), 2.36 (s, 3H, CH_3_), 2.21 (s, 3H, CH_3_). ^13^C NMR (75 MHz, DMSO-d_6_) *δ* ppm 147.3, 142.2, 136.0, 132.0, 131.5, 130.7, 130.6, 128.9, 121.3, 13.4, 11.2. LC/MS (ESI^+^): *m/z* [M + H]^+^ 286.8. Anal. calcd for C_11_H_12_ClN_3_O_2_S (285.75): C, 46.24; H, 4.23; N, 14.71; S, 11.22; found: C, 46.21; H, 4.23; N, 14.72; S, 11.22.

### General procedure 2 (GP2): regiochemically unambiguous preparation of bis-sulfonamides 9 not requiring chromatographic separation regioisomers of bis-sulfonyl chlorides 12

The procedure is analogous to GP1 except for the double amount of chlorosulfonic acid (13.52 g, 116.1 mmol), thionyl chloride (1.52 g, 12.8 mmol) and 25% aqueous ammonia solution (58.0 mmol) used in the preparation.

#### 1-(4-Sulfamoylphenyl)-^1^H-pyrazole-4-sulfonamide (9c)

Prepared from **6a** according to GP2; white solid, m.p. 254–257 °C (*i*-PrOH), yield 56%. ^1^H NMR (400 MHz, DMSO-d_6_) *δ* ppm 9.07 (s, 1H, H_pyrazole_), 8.11 (d, *J* = 8.9 Hz, 2H, H_Ar_), 8.09 (s, 1H, H_pyrazole_), 7.95 (d, *J* = 8.9 Hz, 2H, H_Ar_), 7.45 (m, 4H, 2 x SO_2_NH_2_). ^13^C NMR (75 MHz, DMSO-d_6_) *δ* ppm 143.0, 141.3, 139.9, 129.9, 129.2, 127.8, 119.7. LC/MS (ESI^+^): *m/z* [M + H]^+^ 303.3. Anal. calcd for C_9_H_10_N_4_O_4_S_2_ (302.33): C, 35.76; H, 3.33; N, 18.53; S, 21.21; found: C, 35.71; H, 3.34; N, 18.55; S, 21.23.

#### 3,5-Dimethyl-1-(4-methyl-3-sulfamoylphenyl)-^1^H-pyrazole-4-sulfonamide (9d)

Prepared from **6d** according to GP2; white solid, m.p. 219–221 °C (*i*-PrOH), yield 52%. ^1^H NMR (400 MHz, DMSO-d_6_) *δ* ppm 7.76 (d, *J*_2–6_ = 2.2 Hz, 1H, 2-H_Ar_), 7.31 (dd, *J*_5–6_ = 8.1, Hz, *J*_2–6_ = 2.2 Hz, 1H, 6-H_Ar_), 7.27 (d, *J*_5–6_ = 8.1 Hz, 1H, 5-H_Ar_), 6.48 (br. s., 4H, 2 x SO_2_NH_2_), 2.57 (s, 3H, Ar-CH_3_), 2.35 (s, 3H, CH_3_), 2.28 (s, 3H, CH_3_). ^13^C NMR (75 MHz, DMSO-d_6_) *δ* ppm 147.5, 146.2, 138.7, 136.1, 135.7, 131.9, 125.5, 123.9, 107.9, 20.1, 13.1, 11.8. LC/MS (ESI^+^): *m/z* [M + H]^+^ 345.4. Anal. calcd for C_12_H_16_N_4_O_4_S_2_ (344.41): C, 41.85; H, 4.68; N, 16.27; S, 18.62; found: C, 41.78; H, 4.69; N, 16.21; S, 18.64.

#### 1-(4-Methyl-3-sulfamoylphenyl)-^1^H-pyrazole-4-sulfonamide (9e)

Prepared from **6c** according to GP2; white solid, m.p. 217–220°C (*i*-PrOH), yield 53%. ^1^H NMR (400 MHz, DMSO-d_6_) *δ* ppm 8.97 (s, 1H, H_pyrazole_), 8.35 (d, *J*_2–6_ = 2.4 Hz, 1H, 2-H_A_), 8.06 (s, 1H, H_pyrazole_), 8.02 (dd, *J*_5–6_ = 8.2 Hz, *J*_2–6_ = 2.4 Hz, 1H, 6-H_Ar_), 7.55 (s, 2H, SO_2_NH_2_), 7.54 (d, *J*_5–6_ = 8.2 Hz, 1H, 5-H_Ar_), 7.43 (s, 2H, SO_2_NH_2_), 2.62 (s, 3H, CH_3_). ^13^C NMR (75 MHz, DMSO-d_6_) *δ* ppm 143.8, 139.5, 137.1, 135.4, 134.1, 129.6, 128.8, 122.2, 118.4, 19.7. LC/MS (ESI^+^): *m/z* [M + H]^+^ 317.4. Anal. calcd for C_10_H_12_N_4_O_4_S_2_ (316.36): C, 37.97; H, 3.82; N, 17.71; S, 20.27; found: C, 37.94; H, 3.82; N, 17.67; S, 20.30.

#### 3,5-Dimethyl-1-(3-methyl-4-sulfamoylphenyl)-^1^H-pyrazole-4-sulfonamide (9f)

Prepared from **6f** according to GP2; light grey solid, m.p. 143–146 °C (*i*-PrOH), yield 41%. ^1^H NMR (400 MHz, DMSO-d_6_) *δ* ppm 7.97 (d, *J*_5–6_ = 8.6 Hz, 1H, 5-H_Ar_), 7.54 (m, 4H, 2-H_Ar_, 6-H_Ar_, SO_2_NH_2_), 7.27 (br. s., 2H, SO_2_NH_2_), 2.65 (s, 3H), 2.47 (s, 3H, CH_3_), 2.37 (s, 3H, CH_3_). ^13^C NMR (75 MHz, DMSO-d_6_) *δ* ppm 147.8, 142.2, 141.2, 141.1, 138.2, 128.8, 128.7, 122.9, 122.6, 20.2, 13.4, 12.1. LC/MS (ESI^+^): *m/z* [M + H]^+^ 345.4. Anal. calcd for C_12_H_16_N_4_O_4_S_2_ (344.41): C, 41.85; H, 4.68; N, 16.27; S, 18.62; found: C, 41.82; H, 4.69; N, 16.30; S, 18.63.

#### 1-(3-Methyl-4-sulfamoylphenyl)-^1^H-pyrazole-4-sulfonamide (9g)

Prepared from **6e** according to GP2; white solid, m.p. 205–207 °C (*i*-PrOH), yield 51%. ^1^H NMR (400 MHz, DMSO-d_6_) *δ* ppm 9.05 (s, 1H, H_pyrazole_), 8.07 (s, 1H, H_pyrazole_), 7.98 (d, *J*_2–6_ = 2.0 Hz, 1H, 2-H_Ar_), 7.96 (d, *J*_5–6_ = 8.6 Hz, 1H, 5-H_Ar_), 7.90 (dd, *J*_5–6_ = 8.6 Hz, *J*_2–6_ = 2.0 Hz, 1H, 6-H_Ar_), 7.47 (s, 2H, SO_2_NH_2_), 7.44 (s, 2 H, SO_2_NH_2_), 2.66 (s, 3H, CH_3_). ^13^C NMR (75 MHz, DMSO-d_6_) *δ* ppm 141.2, 141.1, 139.8, 138.6, 129.9, 129.3, 129.1, 122.5, 116.5, 20.4. LC/MS (ESI^+^): *m/z* [M + H]^+^ 317.4. Anal. calcd for C_10_H_11_N_3_O_2_S (316.36): C, 37.97; H, 3.82; N, 17.71; S, 20.27; found: C, 37.94; H, 3.83; N, 17.74; S, 20.29.

#### 3,5-Dimethyl-1-(2-methyl-5-sulfamoylphenyl)-^1^H-pyrazole-4-sulfonamide (9h)

Prepared from **6h** according to GP2; white solid, m.p. 308–310 °C (*i*-PrOH), yield 45%. ^1^H NMR (400 MHz, DMSO-d_6_) *δ* ppm 7.63 (dd, *J*_3–4_ = 7.8 Hz, *J*_4–6_ = 1.3 Hz, 1H, 4-H_Ar_), 7.38 (d, *J*_3–4_ = 7.8 Hz, 1H, 3-H_Ar_), 7.37 (d, *J*_4–6_ = 1.3 Hz, 1H, 6-H_Ar_), 5.96 (br. s., 2H, SO_2_NH_2_), 2.29 (s, 3H, CH_3_), 2.12 (s, 3H, CH_3_), 1.96 (s, 3H, CH_3_); ^13^C NMR (75 MHz, DMSO-d_6_) *δ* ppm 146.8, 145.7, 139.5, 136.2, 136.2, 130.6, 126.8, 124.9, 124.2, 16.7, 12.6, 10.6. LC/MS (ESI^+^): *m/z* [M + H]^+^ 345.4. Anal. calcd for C_12_H_16_N_4_O_4_S_2_ (344.41): C, 41.85; H, 4.68; N, 16.27; S, 18.62; found: C, 41.81; H, 4.68; N, 16.31; S, 18.62.

#### 1-(2-Methyl-5-sulfamoylphenyl)-^1^H-pyrazole-4-sulfonamide (9i)

Prepared from **6g** according to GP2; white solid, m.p. 185–188 °C (*i*-PrOH), yield 47%. ^1^H NMR (400 MHz, DMSO-d_6_) *δ* ppm 8.60 (s, 1H, H_pyrazole_), 8.05 (s, 1H, H_pyrazole_), 7.84 (dd, *J*_3–4_ = 7.8 Hz, *J*_4–6_ = 1.7 Hz, 1H, 4-H_Ar_), 7.80 (d, *J*_4–6_ = 1.3 Hz, 1H, 6-H_Ar_), 7.64 (d, *J*_3–4_ = 7.8 Hz, 1H, 3-H_Ar_), 7.44 (s, 2H, SO_2_NH_2_), 7.41 (s, 2H, SO_2_NH_2_), 2.32 (m, 3H, CH_3_). ^13^C NMR (75 MHz, DMSO-d_6_) *δ* ppm 143.3, 139.1, 137.6, 132.6, 132.5, 128.7, 128.6, 126.3, 123.8, 18.2. LC/MS (ESI^+^): *m/z* [M + H]^+^ 317.4. Anal. calcd for C_10_H_11_N_3_O_2_S (316.36): C, 37.97; H, 3.82; N, 17.71; S, 20.27; found: C, 37.92; H, 3.83; N, 17.75; S, 20.30.

#### 1-(4-Methoxy-3-sulfamoylphenyl)-3,5-dimethyl-^1^H-pyrazole-4-sulfonamide (9j)

Prepared from **6j** according to GP2; white solid, m.p. 254–257°C (*i*-PrOH), yield 79%. ^1^H NMR (400 MHz, DMSO-d_6_) *δ* ppm 7.72 (m, 2H, 2-H_Ar_, 6-H_Ar_), 7.35 (d, *J*_5–6_ = 8.6 Hz, 1H, 5-H_Ar_), 7.29 (s, 2H, SO_2_NH_2_), 7.22 (s, 2H, SO_2_NH_2_), 3.97 (s, 3H, OCH_3_), 2.36 (s, 3H, CH_3_), 2.40 (s, 3H, CH_3_). ^13^C NMR (75 MHz, DMSO-d_6_) *δ* ppm 156.3, 147.4, 141.1, 132.4, 130.9, 130.7, 125.4, 121.8, 113.9, 57.1, 13.4, 11.8. LC/MS (ESI^+^): *m/z* [M + H]^+^ 361.4. Anal. calcd for C_12_H_16_N_4_O_5_S_2_ (360.41): C, 39.99; H, 4.47; N, 15.55; S, 17.79; found: C, 39.94; H, 4.48; N, 15.52; S, 17.81.

#### 1-(3-Methoxy-4-sulfamoylphenyl)-3,5-dimethyl-^1^H-pyrazole-4-sulfonamide (9k)

Prepared from **6l** according to GP2; white solid, m.p. 256–258 °C (*i*-PrOH), yield 82%. ^1^H NMR (400 MHz, DMSO-d_6_) *δ* ppm 7.85 (d, *J*_5–6_ = 8.3 Hz, 1H, 5-H_Ar_), 7.32 (d, *J*_2–6_ = 1.8 Hz, 1H, 2-H_Ar_), 7.28 (s, 2H, SO_2_NH_2_), 7.22 (s, 1H, SO_2_NH_2_), 7.19 (dd, *J*_5–6_ = 8.3 Hz, *J*_2–6_ = 1.8 Hz, 1H, 6-H_Ar_), 3.95 (s, 3H, OCH_3_), 2.50 (s, 3H, CH_3_), 2.36 (m, 3H, CH_3_). ^13^C NMR (75 MHz, DMSO-d_6_) *δ* ppm 157.02, 147.82, 142.86, 141.29, 131.42, 128.96, 122.60, 116.84, 110.34, 57.11, 13.47, 12.15. LC/MS (ESI^+^): *m/z* [M + H]^+^ 361.4.

#### 1-(2-Methoxy-5-sulfamoylphenyl)-3,5-dimethyl-^1^H-pyrazole-4-sulfonamide (9l)

Prepared from **6n** according to GP2; white solid, m.p. 238–242 °C (*i*-PrOH), yield 68%. ^1^H NMR (400 MHz, DMSO-d_6_) *δ* ppm 7.95 (dd, *J*_3–4_ = 8.8 Hz, *J*_4–6_ = 2.4 Hz, 1H, 4-H_Ar_), 7.73 (d, *J*_4–6_ = 2.4 Hz, 1H, 6-H_Ar_), 7.44 (d, *J*_3–4_ = 8.8 Hz, 1H, 3-H_Ar_), 7.36 (s, 2H, SO_2_NH_2_), 7.24 (s, 2H, SO_2_NH_2_), 3.88 (s, 3H, OCH_3_), 2.35 (s, 3H, CH_3_), 2.21 (s, 3H, CH_3_); ^13^C NMR (75 MHz, DMSO-d_6_) *δ* ppm 156.8, 147.5, 142.7, 137.0, 129.1, 127.1, 127.0, 121.3, 113.5, 57.0, 13.4, 11.3. LC/MS (ESI^+^): *m/z* [M + H]^+^ 361.4. Anal. calcd for C_12_H_16_N_4_O_5_S_2_ (360.41): C, 39.99; H, 4.47; N, 15.55; S, 17.79; found: C, 39.91; H, 4.48; N, 15.60; S, 17.82.

#### 1-(4-Chloro-3-sulfamoylphenyl)-3,5-dimethyl-^1^H-pyrazole-4-sulfonamide (9m)

Prepared from **6p** according to GP2; white solid, m.p. 241–243 °C (AcOEt), yield 39%. ^1^H NMR (400 MHz, DMSO-d_6_) *δ* ppm 8.02 (d, *J*_2–6_ = 2.0 Hz, 1H, 2-H_Ar_), 7.80 (m, 4H, 5-H_Ar_, 6-H_Ar_, SO_2_NH_2_), 7.29 (s, 2H, SO_2_NH_2_), 2.47 (s, 3H, CH_3_), 2.37 (m, 3H, CH_3_). ^13^C NMR (75 MHz, DMSO-d_6_) *δ* ppm 148.1, 142.4, 141.4, 137.4, 133.0, 130.4, 129.7, 125.9, 122.7, 13.4, 12.0. LC/MS (ESI^+^): *m/z* [M + H]^+^ 365.8. Anal. calcd for C_11_H_13_ClN_4_O_4_S_2_ (264.83): C, 36.21; H, 3.59; N, 15.36; S, 17.58; found: C, 36.18; H, 3.59; N, 15.33; S, 13.59.

#### 1-(4-Chloro-3-sulfamoylphenyl)-^1^H-pyrazole-4-sulfonamide (9n)

Prepared from **6o** according to GP2; white solid, m.p. 232–235 °C (AcOEt), yield 21%. ^1^H NMR (400 MHz, DMSO-d_6_) *δ* ppm 9.07 (s, 1H, H_pyrazole_), 8.48 (d, *J*_2–6_ = 2.7 Hz, 1H, 2-H_Ar_), 8.15 (dd, *J*_5–6_ = 8.8 Hz, *J*_2–6_ = 2.7 Hz, 1H, 6-H_Ar_), 8.09 (s, 1H, H_pyrazole_), 7.81 (d, *J*_5–6_ = 8.8 Hz, 1H, 5-H_Ar_), 7.79 (s, 2H, SO_2_NH_2_), 7.47 (s, 2H, SO_2_NH_2_). ^13^C NMR (75 MHz, DMSO-d_6_) *δ* ppm 142.7, 139.9, 137.8, 133.3, 130.1, 129.2, 129.1, 123.4, 120.0. LC/MS (ESI^+^): *m/z* [M + H]^+^ 337.8. Anal. calcd for C_9_H_9_ClN_4_O_4_S_2_ (336.78): C, 32.10; H, 2.69; N, 16.64; S, 19.04; found: C, 32.05; H, 2.70; N, 16.69; S, 19.05.

#### 1-(3-Chloro-4-sulfamoylphenyl)-3,5-dimethyl-^1^H-pyrazole-4-sulfonamide (9o)

Prepared from **6r** according to GP2; white solid, m.p. 198–201 °C (AcOEt), yield 43%. ^1^H NMR (300 MHz, DMSO-d_6_) *δ* ppm 7.86 (d, *J*_2–6_ = 2.4 Hz, 1H, X-H_Ar_), 7.80 (d, *J*_5–6_ = 8.6 Hz, 1H, A-H_Ar_), 7.54 (dd, *J*_5–6_ = 8.6, *J*_2–6_ = 2.4 Hz, 1H, B-H_Ar_), 7.29 (s, 2H, in exchange, SO_2_NH_2_), 2.46 (s, 3H, CH_3_), 2.36 (s, 3H, CH_3_). ^13^C NMR (75 MHz, DMSO-d_6_) *δ* ppm 147.9, 141.3, 138.5, 132.2, 131.6, 131.5, 127.5, 125.9, 122.5, 13.4, 11.9. LC/MS (ESI^+^): *m/z* [M + H]^+^ 365.8. Anal. calcd for C_11_H_13_ClN_4_O_4_S_2_ (264.83): C, 36.21; H, 3.59; N, 15.36; S, 17.58; found: C, 36.19; H, 3.59; N, 15.35; S, 13.58.

#### 1-(3-Chloro-4-sulfamoylphenyl)-^1^H-pyrazole-4-sulfonamide (9p)

Prepared from **6q** according to GP2; white solid, m.p. 195–197 °C (AcOEt), yield 25%. ^1^H NMR (400 MHz, DMSO-d_6_) *δ* ppm 9.07 (s, 1H, H_pyrazole_), 8.23 (d, *J*_2–6_ = 2.3 Hz, 1H, 2-H_Ar_), 8.06 (s, 1H, H_pyrazole_), 7.78 (dd, *J*_5–6_ = 8.6 Hz, *J*_2–6_ = 2.3 Hz, 1H, 6-H_Ar_),7.94 (d, *J*_5–6_ = 8.6 Hz, 1H, 5-H_Ar_), 7.42 (s, 4H, 2 x SO_2_NH_2_). ^13^C NMR (75 MHz, DMSO-d_6_) *δ* ppm 139.8, 138.9, 132.6, 132.0, 130.1, 129.9, 129.3, 121.2, 119.5. LC/MS (ESI^+^): *m/z* [M + H]^+^ 337.8. Anal. calcd for C_9_H_9_ClN_4_O_4_S_2_ (336.78): C, 32.10; H, 2.69; N, 16.64; S, 19.04; found: C, 32.07; H, 2.69; N, 16.66; S, 19.04.

#### 1-(2-Chloro-5-sulfamoylphenyl)-3,5-dimethyl-^1^H-pyrazole-4-sulfonamide (9q)

Prepared from **6t** according to GP2; white solid, m.p. 230–232 °C (AcOEt), yield 41%. ^1^H NMR (400 MHz, DMSO-d_6_) *δ* ppm 8.00 (dd, *J*_3–4_ = 8.3 Hz, *J*_4–6_ = 1.7 Hz, 1H, 4-H_Ar_), 7.94 (d, *J*_3–4_ = 8.3 Hz, 1H, 3-H_Ar_), 7.91 (d, *J*_4–6_ = 1.7 Hz, 1H, 6-H_Ar_), 7.57 (s, 2H, SO_2_NH_2_), 7.28 (s, 2H, SO_2_NH_2_), 2.38 (s, 3H, CH_3_), 2.26 (s, 3H, CH_3_). ^13^C NMR (75 MHz, DMSO-d_6_) *δ* ppm 148.1, 144.6, 142.5, 136.3, 135.0, 131.8, 128.9, 127.7, 121.8, 13.4, 11.3. LC/MS (ESI^+^): *m/z* [M + H]^+^ 365.8. Anal. calcd for C_11_H_13_ClN_4_O_4_S_2_ (264.83): C, 36.21; H, 3.59; N, 15.36; S, 17.58; found: C, 36.15; H, 3.60; N, 15.29; S, 13.60.

#### 1-(2-Chloro-5-sulfamoylphenyl)-^1^H-pyrazole-4-sulfonamide (9r)

Prepared from **6s** according to GP2; white solid, m.p. 235–237 °C (AcOEt), yield 31%. ^1^H NMR (400 MHz, DMSO-d_6_) *δ* ppm 8.71 (s, 1H, H_pyrazole_), 8.10 (s, 1H, H_pyrazole_), 8.00 (m, 1H, H_Ar_), 7.94 (m, 2H, H_Ar_), 7.39 (s, 4H, 2 x SO_2_NH_2_). ^13^C NMR (75 MHz, DMSO-d_6_) *δ* ppm 140.1, 139.4, 132.8, 132.4, 130.1, 130.1, 128.9, 120.9, 118.9. LC/MS (ESI^+^): *m/z* [M + H]^+^ 337.8. Anal. calcd for C_9_H_9_ClN_4_O_4_S_2_ (336.78): C, 32.10; H, 2.69; N, 16.64; S, 19.04; found: C, 32.05; H, 2.70; N, 16.69; S, 19.05.

### General procedure 3 (GP3): preparation of mono-sulfonamides 7–8 requiring interim chromatographic separation of regioisomeric mono-sulfonyl chlorides 10–11

The procedure is analogous to GP1 except for after evaporation of chloroform, the mixture of regioisomeric mono-sulfonyl chlorides was fractionated on silica gel using an appropriate gradient of ethyl acetate in hexanes as eluent, fractions containing different isomers of mono-sulfonylchlorides were pooled separately, concentrated *in vacuo* and then, also separately, converted to respective mono-sulfonamides (by treatment with 25% aqueous ammonia) which were characterized.

#### 4-(3,5-Dimethyl-^1H^-pyrazol-1-yl)-2-methylbenzenesulfonamide (7c)

Prepared (along with **8d**) from **6f** according to GP3; white solid, m.p. 194–196 °C (AcOEt), yield 35%. ^1^H NMR (400 MHz, DMSO-d_6_) *δ* ppm 7.92 (d, *J*_5–6_ = 8.2 Hz, 1H, 6-H_Ar_), 7.55 (d, *J*_3–5_ = 2.4 Hz, 1H, 3-H_A_), 7.50 (dd, *J*_5–6_ = 8.2 Hz, *J*_3–5_ = 2.4 Hz, 1H, 5-H_Ar_), 7.45 (br. s., 2H, SO_2_NH_2_), 6.11 (s, 1H, H_pyrazole_), 2.64 (s, 3 H, CH_3_), 2.35 (s, 3H, CH_3_), 2.18 (s, 3H, CH_3_). ^13^C NMR (75 MHz, DMSO-d_6_) *δ* ppm 149.1, 142.4, 140.5, 140.0, 137.8, 128.6, 127.0, 120.9, 108.5, 20.2, 13.7, 12.8. LC/MS (ESI^+^): *m/z* [M + H]^+^ 266.3. Anal. calcd for C_12_H_15_N_3_O_2_S (265.34): C, 54.32; H, 5.70; N, 15.84; S, 12.08; found: C, 54.30; H, 5.70; N, 15.81; S, 12.10.

#### 3-(3,5-Dimethyl-^1H^-pyrazol-1-yl)-4-methylbenzenesulfonamide (7e)

Prepared (along with **8e**) from **6h** according to GP3; white solid, m.p. 112–114 °C (*i*-PrOH), yield 33%; ^1^H NMR (300 MHz, DMSO-d_6_) *δ* ppm 7.89 (dd, *J*_5–6_ = 8.0 Hz, *J*_2–6_ = 1.5 Hz, 1H, 6-H_Ar_), 7.67 (d, *J*_2–6_ = 1.5 Hz, 1H, 2-H_Ar_), 7.65 (d, *J*_5–6_ = 7.8 Hz, 1H, 5-H_Ar_), 7.44 (br. s., 2H, SO_2_NH_2_), 7.24 (s, 2H, SO_2_NH_2_), 2.38 (s, 3H, CH_3_), 2.23 (s, 3H, CH_3_), 2.08 (s, 3H, CH_3_). ^13^C NMR (75 MHz, DMSO-d_6_) *δ* ppm 147.4, 143.3, 141.8, 140.2, 137.6, 132.3, 127.0, 125.5, 121.3, 17.3, 13.4, 11.3. LC/MS (ESI^+^): *m/z* [M + H]^+^ 266.3. Anal. calcd for C_12_H_15_N_3_O_2_S (265.34): C, 54.32; H, 5.70; N, 15.84; S, 12.08; found: C, 54.27; H, 5.70; N, 15.86; S, 12.09.

#### 2-Chloro-4-(^1^H-pyrazol-1-yl)benzenesulfonamide (7m)

Prepared (along with **8h**) from **6q** according to GP3; white solid, m.p. 181–183 °C, yield 17%. ^1^H NMR (300 MHz, DMSO-d_6_) *δ* ppm 8.68 (d, *J* = 2.31 Hz, 1H, H_pyrazole_), 8.15 (d, *J*_3–5_ = 2.0 Hz, 1H, 3-H_Ar_), 8.08 (d, *J*_5–6_ = 8.7 Hz, 1H, 6-H_Ar_), 8.00 (dd, *J*_5–6_ = 8.7 Hz, *J*_3–5_ = 2.0 Hz, 1H, 5-H_Ar_), 7.84 (d, *J* = 1.32 Hz, 1H, H_pyrazole_), 7.66 (s, 2H, SO_2_NH_2_), 6.63 (m, 1H, H_pyrazole_). ^13^C NMR (75 MHz, DMSO-d_6_) *δ* ppm 142.9, 142.8, 138.5, 132.2, 130.9, 129.2, 120.7, 116.8, 109.6. LC/MS (ESI^+^): *m/z* [M + H]^+^ 258.7. Anal. calcd for C_9_H_8_ClN_3_O_2_S (257.70): C, 41.95; H, 3.13; N, 16.31; S, 12.44; found: C, 41.88; H, 3.13; N, 16.29; S, 12.46.

#### 3,5-Dimethyl-1-(3-methylphenyl)-^1^H-pyrazole-4-sulfonamide (8d)

Prepared (along with **7c**) from **6f** according to GP3; white solid, m.p. 174–176 °C (AcOEt), yield 34%. ^1^H NMR (400 MHz, DMSO-d_6_) *δ* ppm 7.42 (t, *J* = 7.9 Hz, 1H, H_Ar_), 7.28 (m, 3H, H_Ar_), 7.22 (s, 2H, SO_2_NH_2_), 2.40 (s, 3H, CH_3_), 2.38 (s, 3H, CH_3_), 2.35 (s, 3H, CH_3_). ^13^C NMR (75 MHz, DMSO-d_6_) *δ* ppm 147.1, 140.7, 139.5, 138.8, 129.6, 129.5, 126.4, 122.9, 121.8, 21.2, 13.4, 11.9. LC/MS (ESI^+^): *m/z* [M + H]^+^ 266.3. Anal. calcd for C_12_H_15_N_3_O_2_S (265.34): C, 54.32; H, 5.70; N, 15.84; S, 12.08; found: C, 54.28; H, 5.70; N, 15.79; S, 12.09.

#### 3,5-Dimethyl-1-(2-methylphenyl)-^1^H-pyrazole-4-sulfonamide (8e)

Prepared (along with **7e**) from **6h** according to GP3; white solid, m.p. 161–163°C, yield 88%. ^1^H NMR (400 MHz, DMSO-d_6_) *δ* ppm 7.45 (m, 2H, H_Ar_), 7.37 (t, *J* = 7.0 Hz, 1H, H_Ar_), 7.26 (d, *J* = 7.8 Hz, 1H, H_Ar_), 7.21 (s, 1H, SO_2_NH_2_), 2.35 (s, 3H, CH_3_), 2.17 (s, 3H, CH_3_), 1.96 (s, 3H, CH_3_); ^13^C NMR (75 MHz, DMSO-d_6_) *δ* ppm 146.8, 141.5, 137.6, 135.9, 131.7, 130.2, 128.2, 127.4, 120.7, 17.2, 13.5, 11.3. LC/MS (ESI^+^): *m/z* [M + H]^+^ 266.3. Anal. calcd for C_12_H_15_N_3_O_2_S (265.34): C, 54.32; H, 5.70; N, 15.84; S, 12.08; found: C, 54.26; H, 5.70; N, 15.81; S, 12.08.

#### (3-Chlorophenyl)-^1^H-pyrazole-4-sulfonamide (8h)

Prepared (along with **7m**) from **6q** according to GP3; white solid, m.p. 82–83 °C, yield 31%. ^1^H NMR (300 MHz, DMSO-d_6_) *δ* ppm 11.63 (br s, 2H, in exchange, SO_2_NH_2_), 8.61 (s, 1H, H_pyrazole_), 7.96 (s, 1H, H_Ar_), 7.85 (d, *J* = 8.9 Hz, 1H, H_Ar_), 7.72 (s, 1H, H_pyrazole_), 7.49 (t, *J* = 8.1 Hz, 1H, H_Ar_), 7.34 (d, *J* = 7.9 Hz, 1H, H_Ar_); ^13^C NMR (75 MHz, DMSO-d_6_) *δ* ppm 141.0, 139.8, 134.4, 133.5, 131.6, 126.8, 126.5, 118.6, 117.3. LC/MS (ESI^+^): *m/z* [M + H]^+^ 258.7. Anal. calcd for C_9_H_8_ClN_3_O_2_S (257.70): C, 41.95; H, 3.13; N, 16.31; S, 12.44; found: C, 41.91; H, 3.13; N, 16.34; S, 12.44.

### General procedure 4 (GP4): preparation of bis-sulfonamides 9 requiring interim chromatographic separation of regioisomeric bis-sulfonyl chlorides 12

The procedure is analogous to GP2 except for after evaporation of chloroform, the mixture of regioisomeric bis-sulfonyl chlorides was fractionated on silica gel using an appropriate gradient of ethyl acetate in hexanes as eluent, fractions containing different isomers of bis-sulfonylchlorides were pooled separately, concentrated *in vacuo* and then, also separately, converted to respective bis-sulfonamides (by treatment with 25% aqueous ammonia) which were characterized.

#### 3,5-Dimethyl-1-(3-sulfamoylphenyl)-^1^H-pyrazole-4-sulfonamide (9a)

Prepared (along with **9b**) from **6b** according to GP4; white solid, m.p. 223–225 °C (*i*-PrOH), yield 89%. ^1^H NMR (400 MHz, DMSO-d_6_) *δ* ppm 7.90 (m, 2H, H_Ar_), 7.77 (m, 2H, H_Ar_), 7.53 (s, 2H, SO_2_NH_2_), 7.28 (s, 2H, SO_2_NH_2_), 2.47 (s, 3H, CH_3_), 2.38 (s, 3H, CH_3_). ^13^C NMR (75 MHz, DMSO-d_6_) *δ* ppm 147.9, 145.8, 141.2, 139.0, 130.7, 128.6, 125.7, 122.9, 122.5, 13.4, 12.0. LC/MS (ESI^+^): *m/z* [M + H]^+^ 331.4. Anal. calcd for C_11_H_14_N_4_O_4_S_2_ (330.39): C, 39.99; H, 4.27; N, 16.96; S, 19.41; found: C, 39.97; H, 4.27; N, 17.00; S, 19.43.

#### 3,5-Dimethyl-1-(4-sulfamoylphenyl)-^1^H-pyrazole-4-sulfonamide (9b)

Prepared (along with **9a**) from **6b** according to GP4; white solid, m.p. 272–274 °C (i-PrOH), yield 92%. ^1^H NMR (400 MHz, DMSO-d_6_) *δ* ppm 7.98 (d, *J* = 8.6 Hz, 2H, H_Ar_), 7.74 (d, *J* = 8.6 Hz, 2H, H_Ar_), 7.52 (s, 2H, SO_2_NH_2_), 7.30 (s, 2H, SO_2_NH_2_), 2.51 (s, 3H, CH_3_), 2.38 (s, 3H, CH_3_). ^13^C NMR (75 MHz, DMSO-d_6_) *δ* ppm 147.9, 144.0, 141.2, 127.3, 125.9, 122.6, 13.5, 12.1. LC/MS (ESI^+^): *m/z* [M + H]^+^ 331.3. Anal. calcd for C_11_H_14_N_4_O_4_S_2_ (330.39): C, 39.99; H, 4.27; N, 16.96; S, 19.41; found: C, 39.93; H, 4.28; N, 17.00; S, 19.42.

#### 4-(3,5-Dimethyl-^1^H-pyrazol-1-yl)benzenesulfonamide (7o)

A mixture of 4-hydrazinobenzene sulfonamide hydrochloride (10.0 mmol) and acetylacetone (10 mmol) in ethanol (10 ml) was heated at reflux for 90 min and then cooled down to 0 °C. The precipitate formed was collected by filtration and washed with cold ethanol (5 ml). Crystallization from EtOH afforded the title compound in 82% yield.

^1^H NMR (400 MHz, DMSO-d_6_) *δ* ppm 7.91 (d, *J* = 8.6 Hz, 2H, H_Ar_), 7.72 (d, *J* = 8.6 Hz, 2H, H_Ar_), 7.43 (br. s., 2H, SO_2_NH_2_), 6.13 (s, 1H, H_pyrazole_), 2.36 (s, 3H, CH_3_), 2.19 (s, 3H, CH_3_). ^13^C NMR (75 MHz, DMSO-d_6_) *δ* ppm 149.3, 142.3, 140.2, 127.2, 124.0, 108.7, 13.7, 12.8. LC/MS (ESI^+^): *m/z* [M + H]^+^ 252.3. Anal. calcd for C_11_H_13_N_3_O_2_S (251.31): C, 52.57; H, 5.21; N, 16.72; S, 12.76; found: C, 52.55; H, 5.21; N, 16.70; S, 12.77.

### Docking studies

The crystal structure of *h*CA I (pdb code 1AZM^11^), *h*CA II (pdb code 2AW1^12^), *h*CA IV (pdb code 1ZNC^13^), and *h*CA VII (pdb code 3ML5^14^) was taken from the Protein Data Bank^15^. After adding hydrogen atoms and removing complexed ligands, the four proteins were minimized using Amber 14 software^16^ and parm03 force field at 300 K. The four proteins were placed in a rectangular parallelepiped water box, an explicit solvent model for water, TIP3P, was used and the complexes were solvated with a 20 Å water cap. Sodium ions were added as counter ions to neutralize the system. Two steps of minimization were then carried out; in the first stage, we kept the protein fixed with a position restraint of 500 kcal/mol Å^2^ and we solely minimized the positions of the water molecules. In the second stage, we minimized the entire system through 5000 steps of steepest descent followed by conjugate gradient (CG) until a convergence of 0.05 kcal/Å•mol. Automated docking was carried out by means of the AUTODOCK 4.2 program^17^ using the improved force field^18^. Autodock Tools was used in order to identify the torsion angles in the ligand, add the solvent model and assign the Kollman atomic charges to the protein. The ligand charge was calculated using the Gasteiger method. The sulfonamide group involved in the interaction with the Zinc ion was considered as deprotonated, as reported in literature^19^^,^^20^. A grid spacing of 0.375 Å and a distance-dependent function of the dielectric constant were used for the energetic map calculations. Using the Lamarckian Genetic Algorithm, the docked compounds were subjected to 100 runs of the Autodock search, using 500,000 steps of energy evaluation and the default values of the other parameters. Cluster analysis was performed on the results using an RMS tolerance of 2.0 Å and the best docked conformations were taken into account.

### Carbonic anhydrase inhibition assay

An applied photophysics stopped-flow instrument has been used for assaying the CA catalysed CO_2_ hydration activity^21^. Phenol red (at a concentration of 0.2 mM) has been used as indicator, working at the absorbance maximum of 557 nm, with 20 mM Tris (pH 8.3) as buffer, and 20 mM Na_2_SO_4_ (for maintaining constant the ionic strength), following the initial rates of the CA-catalysed CO_2_ hydration reaction for a period of 10–100 s. The CO_2_ concentrations ranged from 1.7 to 17 mM for the determination of the kinetic parameters and inhibition constants. For each inhibitor at least six traces of the initial 5–10% of the reaction have been used for determining the initial velocity. The uncatalysed rates were determined in the same manner and subtracted from the total observed rates. Stock solutions of inhibitor (0.1 mM) were prepared in distilled-deionized water and dilutions up to 0.005 nM were done thereafter with the assay buffer. Inhibitor and enzyme solutions were preincubated together for 15 min at room temperature prior to assay, in order to allow for the formation of the E–I complex. The inhibition constants were obtained by non-linear least-squares methods using PRISM 3 and the Cheng–Prusoff equation, as reported earlier, and represent the mean from at least three different determinations. All CA isoforms were recombinant ones obtained in-house^22–25^.

## Results and discussion

### Compound synthesis

**Scheme 1.**
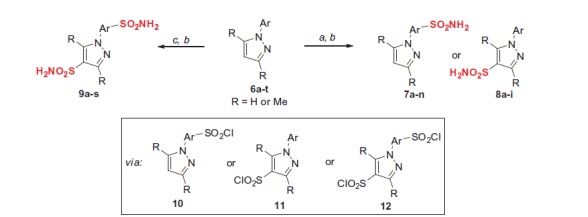
Mono- and bis-sulfonamide synthesis via direct sulfochlorination of **6a–t**. *Reagents and conditions:* (a) ClSO_3_H (10 equiv.), SOCl_2_ (1.1 equiv.), 10–70 °C, 1–24 h; (b) aq. NH_3_ (20 equiv.), acetone, 50 °C, 1 h; (c) ClSO_3_H (20 equiv.), SOCl_2_ (2.2 equiv.), 70–120 °C, 7–48 h.

In order to create and investigate a set of the required compounds for this study, we began by performing synthesis of mono-sulfonamides **7a–n** or **8a–i** as well as of bis-sulfonamides **9a–s** by direct sulfochlorination of a large set of N-arylpyrazoles **6a–t** (all of which are known compounds and/or can be prepared according to straightforward technique^26^), followed by conversion of the respective mono-and bis-sulfochlorites **9, 10** and **11** on treatment with aqueous ammonia (Scheme 1).

The regiospecificity of the sulfochlorination was unequivocally established for every substrate **6a–t** by means for correlational NOESY spectroscopy (ESI) and is depicted in Figure 4 in a straightforward fashion.

On close observation, it is the relative stereoelectronic character of the phenyl vs. the pyrazole unit that governed the direction of the first sulfochlorination. With some exceptions, dimethylpyrazole unit was the first affected with electron-neutral or moderately electron-rich aryls. There are a few mixed situations (**6f** and **6h**). Clearly, introduction of an anisyl group swayed the sulfochlorination completely to that group and made it impossible to produce bis-sulfonamides from those compounds (leading to only tar formation when attempting (**6i–k**, **6m**). Some compounds (**6f**, **6h**, **6q**) allowed making mono-sulfonamides at both the phenyl and the pyrazoles portions of the molecule, thereby contributing even more to the diversity of this *h*CA-probing set compounds. Altogether, to the best of our knowledge, the one presented in Figure 4 is the most comprehensive mono- and bis-sulfochlorination match presented to-date. Of course, some “arrows” require rather mild conditions to realize; others – a lot more forcing conditions, particularly when it comes to achieving the second sulfochlorination. For clarity of the presentation in Figure 4, the reader is referred to Tables 1–3 and the ESI for specific reaction times and temperatures.

**Scheme 2.**
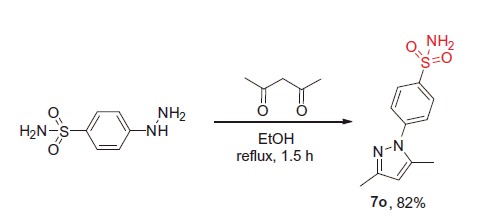
Preparation of compound **7o**.

Compound **7o** which constitutes an important SAR point could not be prepared as described in Scheme 1, was prepared by a direct route^26^ shown in Scheme 2.

### Biological activity

The inhibitory profile obtained for mono-sulfonamides **7a–o** in a stopped-flow kinetics assay against human CA I, II, IV and VII is shown in Table 1.

Several observations emerge from the data in Table 1. Clearly, some respectable *h*CA II levels are achievable. Compounds **7c** and **7o** are distinctly acetazolamide-like. The difficult-to-inhibit hCA IV is not giving high inhibition results throughout, considering the “detrimental methoxy” phenomenon present in **7g**–**7l** (previously noted by us^10^ and tentatively justified). When it comes to *h*CA IV isoform, some striking restoration of potency is observed in **7b** (on top of a high selectivity) and, particularly, in **7n** (where overall selectivity is not that good). The *h*CA VII selectivity of compound **7j** is also quite notable (and was not ablated, in this case, by the “detrimental methoxy” phenomenon). Altogether, from this set alone, compounds **7b** and **7j** can be developed as selective probes for *h*CA IV and VII, respectively.

The set of compounds presented in Table 2 is marked by a virtual absence of *h*CA IV activity. The compounds can be regarded as weaker, nonselective analogues of acetazolamide and are primarily of interest as a reference set to compare with the bis-sufonamide set discussed below.

Among bis-sulfonamides **9a–r**, several instances of restoring specific inhibitory potencies (compared to the respective mono-sulfonamide parts **7** or **8**) can be noted, which is suggestive of a possibility of alternative binding mode compared to either **7** or **8**. Most notable example is provided by compound **9l** whose analogue **7k** was inactive throughout the panel (most likely, due to the “detrimental methoxy” effect noted earlier). However, the potency is restored against three isoforms in **9l**, which is indicative of the inhibitor’s binding to the target at the pyrazole sulfonamide portion.

Notable examples of isoform selectivity identified within **9a–r** set include: **9d** (selective *h*CA IV inhibitor); **9h** (selective inhibitor of *h*CA I) which, *cf.***7f**, demonstrates the power of an additional sulfonamide in ablating activity against all other isoforms; **9q** (selective *h*CA II inhibitor).

### *In silico* modelling

**Figure 5.**
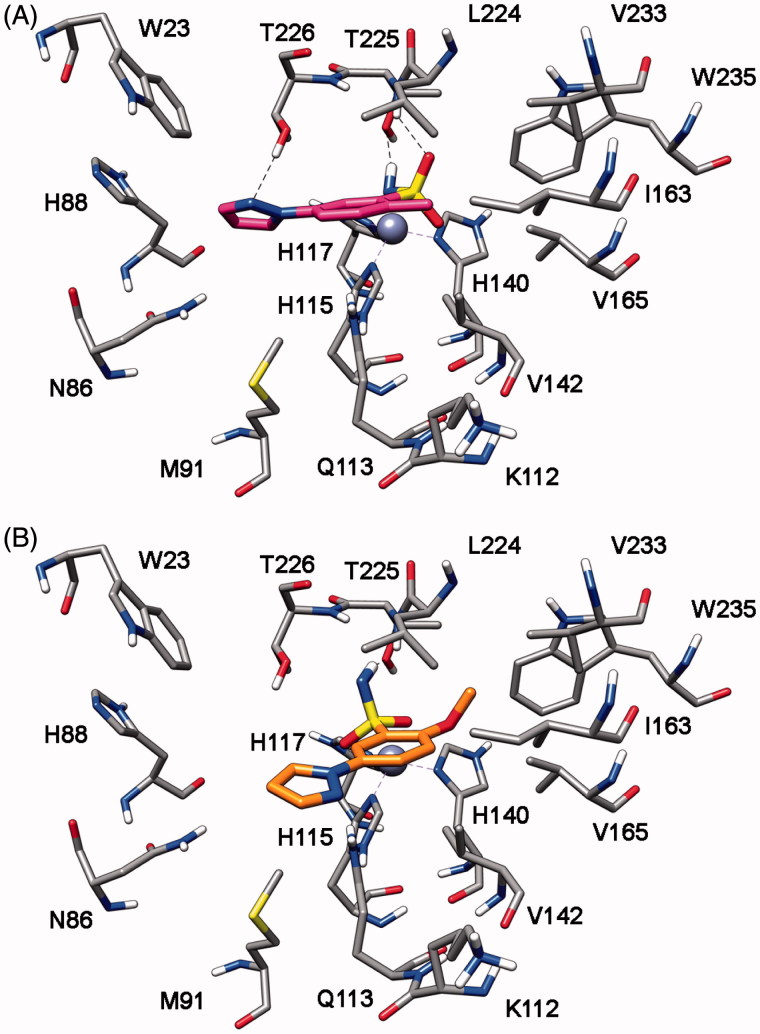
Docking of compound **7b** (A) and **7h** (B) into *h*CA IV.

**Figure 6.**
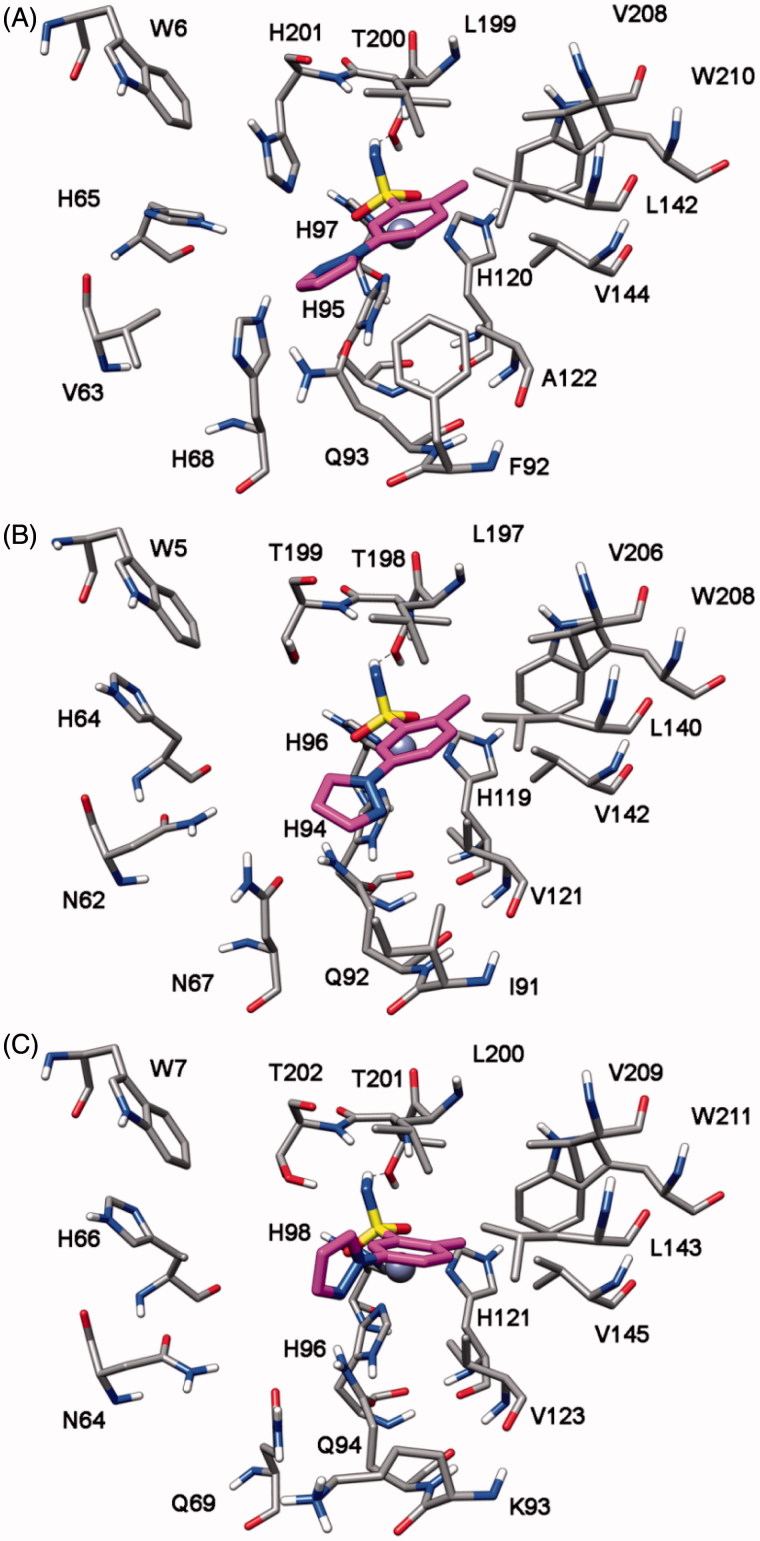
Docking of compound **7b** into *h*CA I (A), *h*CA II (B) and *h*CA VII (C).

In order to identify the possible binding mode of the new mono- and bis-sulfonamides disclosed herein and also rationalize the SAR trends observed, representative compounds were docked into the *h*CA I, II, IV and VII X-ray structures. Figure 5(A) shows the docking of compound **7b** into the active site of *h*CA IV. The sulfonamide group acts as a zinc binding group (ZBG) and forms hydrogen bonds with the protein backbone and the hydroxy group of T225; the phenyl ring does not show important lipophilic interactions whereas the methyl substituent in inserted into a lipophilic cleft mainly delineated by V142, I163, L224 and V233. With regards to the pyrazole ring, it points towards H88 and shows an H-bond with T226. The docking analysis of this compound into *h*CA I, II and VII highlights a completely different binding mode for these three enzymes. As shown in Figure 6, in all three cases the pyrazole ring points towards the entrance of the binding site and the phenyl ring shows lipophilic interactions with V121, V142 and L197 (*h*CA II numbering). The sulfonamide group acts as a ZBG with an uncommon coordination, with one of the two oxygens that coordinates the zinc ion and the nitrogen that forms an H-bond T198. This different binding disposition is in agreement with the selectivity profile of this compound (potent inhibition of *h*CA IV with virtually no activity against the other three isoforms) and could be due to small differences in the lipophilic cleft in which the methyl group interacts in *h*CA IV, as this enzyme exhibits the non-conserved I163 that is substituted in the other three CA subtypes by a Leucine residue. The substitution of the methyl with a methoxy substituent in the benzene ring (as in compound **7h**) triggers the loss of *h*CA IV inhibition activity. As shown in Figure 5(B), the methoxy group is not able to interact into the lipophilic cleft mainly delineated by V142, I163, L224 and V233 and for this reason this compound shows a binding disposition very similar to that observed for compound **7b** into *h*CA I, II and VII (Figure 6). The pyrazole ring points towards the entrance of the binding site, the phenyl ring shows lipophilic interactions with V142, I163, V165 and L224 whereas one of the two oxygens of the sulfonamide group coordinates the zinc ion and the amide nitrogen forms an H-bond T225.

**Figure 7.**
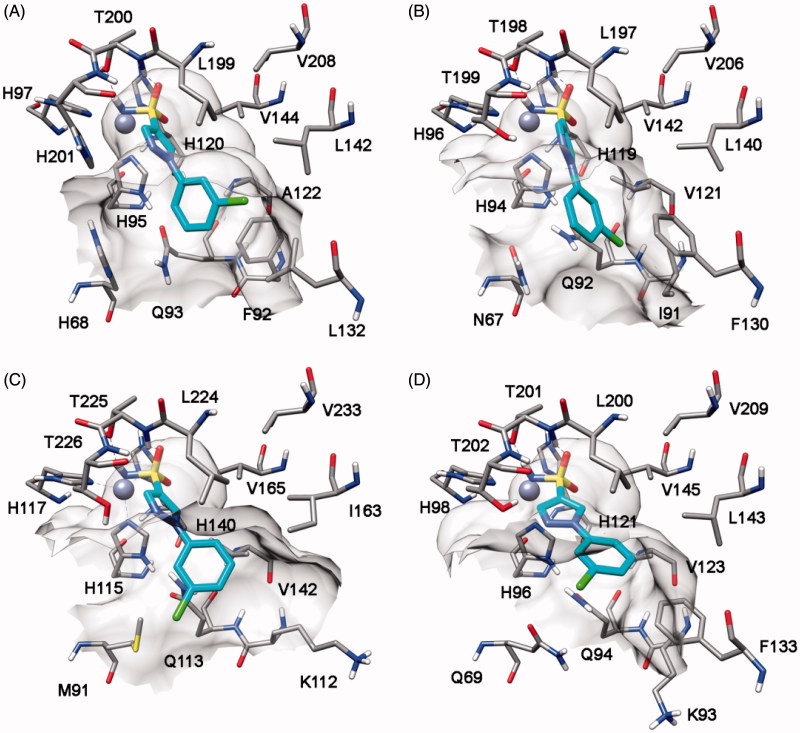
Docking of compound **8h** into *h*CA I (A), *h*CA II (B), *h*CA IV (C) and *h*CA VII (D).

Compound **8h** highlights a good *h*CA I, II and VII inhibition activity with selectivity against *h*CA IV. Docking studies suggests that this compound interacts with a similar binding disposition into the four different CA subtypes. The sulfonamide group acts as the ZBG and forms hydrogen bonds with the protein backbone and the hydroxy group of T198 (*h*CA II numbering), the pyrazole ring shows a lipophilic interaction with the conserved L197 whereas the chlorophenyl group shows lipophilic interactions with the conserved V121, L140 and L197. Furthermore, this aromatic ring shows a lipophilic interaction with F130 (L132 for *h*CA I and F133 for *h*CA VII) that partially occludes the binding site cavity (see Figure 7). In *h*CA IV this residue is substituted by an asparagine residue and corresponds to a region that in *h*CA IV is far away from the binding site, thus leaving the chlorophenyl ring more exposed to the solvent (Figure 7).

**Figure 8.**
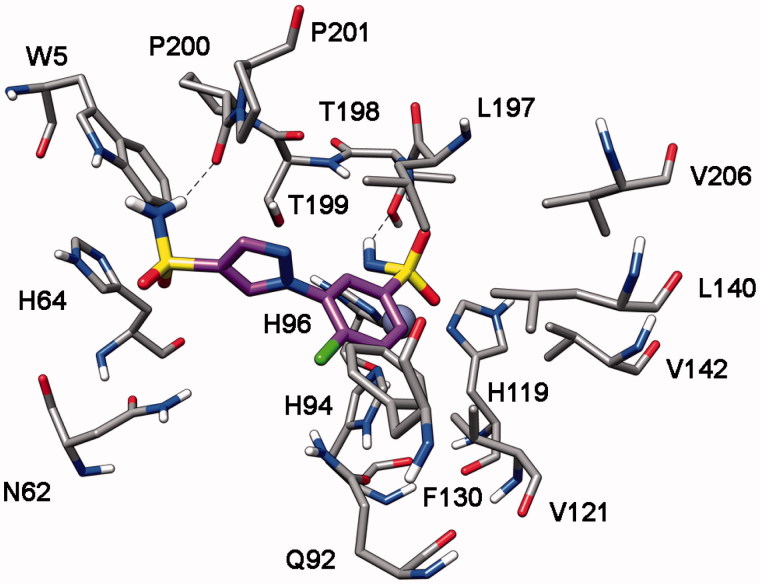
Docking of compound **9r** into *h*CA II.

Finally, the analysis of the docking results for compound **9r** suggests that in the four CA subtypes the sulfonamide group attached to the *o*-chlorophenyl fragment act as the ZBG. The phenylpyrazole portion shows lipophilic interactions with F130, L140, L197, P201 (*h*CA II numbering), and the sulfonamide group attached to the pyrazole ring forms an H-bond with the oxygen backbone of P200 (Figure 8).

## Conclusions

In this work, we systematically harnessed the power of direct sulfochlorination of a series of known, diversely substituted N-arylpyrazole to arrive at three distinct series of compounds. In each series, SAR generalizations have been made and a number of selective compounds (working against only one target in the panel of four or having high selectivity indices) have been identified. The observed selectivity patterns have been rationalized by modelling. The compounds thus identified can serve as isoform-selective tool inhibitors to probe for cellular processes and their linkage to particular *h*CA isoforms.
